# Peripheral hematological inflammatory signatures in the amyloid-defined Alzheimer’s disease continuum: associations with disease stage, cognitive function, and astroglial activation

**DOI:** 10.3389/fimmu.2026.1959479

**Published:** 2026-10-14

**Authors:** Ying He, Jingyu Zhou, Xiaolei Wu, Jing Sheng, Ziyi Zhang, Zijuan Feng, Shuting Zhang

**Affiliations:** 1 Department of Neurology, West China Hospital, Sichuan University, Chengdu, China; 2 Department of Cancer Center, Army Medical Center of PLA (Daping Hospital), Army Medical University, Chongqing, China

**Keywords:** Alzheimer’s disease continuum, blood-based biomarkers, cognitive impairment, hematological inflammatory indices, peripheral inflammation

## Abstract

**Background:**

Peripheral immune-inflammatory dysregulation has been implicated in Alzheimer’s disease (AD) and cognitive decline. However, whether routinely available hematological inflammatory indices are associated with biological and clinical stages across the AD continuum and provide incremental information beyond established risk factors remains unclear. We aimed to investigate the associations of neutrophil-to-lymphocyte ratio (NLR), systemic immune-inflammation index (SII), and hemoglobin-to-red blood cell distribution width ratio (HRR) with AD continuum stage, cognition, and AD biomarkers.

**Methods:**

This cross-sectional study included 267 participants who underwent comprehensive cognitive assessment, peripheral blood testing, and amyloid-β positron emission tomography (Aβ-PET) imaging. Participants were categorized into three groups according to cognitive status and available amyloid biomarker profiles: cognitively unimpaired (CU-Aβ^-^), amyloid-positive mild cognitive impairment (MCI-Aβ^+^), and amyloid-positive Alzheimer’s disease dementia (AD-Aβ^+^). Hematological inflammatory indices, including neutrophil-to-lymphocyte ratio (NLR), systemic immune-inflammation index (SII), and hemoglobin-to-red cell distribution width ratio (HRR), were calculated from routine blood parameters. A composite inflammatory index (Inflam_PC1) was derived using principal component analysis. Ordinal logistic regression models were used to examine the associations between inflammatory indices and AD continuum stage after adjustment for demographic, vascular, metabolic, and genetic factors.

**Results:**

Inflammatory profiles differed across the AD continuum, with increased NLR and SII and decreased HRR levels in more advanced stages. Higher NLR (OR = 1.54, 95% CI: 1.25-1.91, *P* < 0.001) and SII (OR = 1.29 per 100-unit increase, 95% CI: 1.15-1.45, *P* < 0.001) were independently associated with advanced AD continuum stages, whereas HRR was not. The composite inflammatory signature showed the strongest association (OR = 1.63 per SD increase, 95% CI: 1.30-2.03, *P* < 0.001) and provided incremental statistical improvement beyond conventional risk factors. Higher inflammatory burden was associated with poorer cognitive and functional performance and correlated with plasma glial fibrillary acidic protein (GFAP) level.

**Conclusions:**

Peripheral hematological inflammatory signatures were associated with Alzheimer’s disease continuum. Higher NLR and SII levels and a composite inflammatory signature were independently associated with more advanced disease stages, cognitive impairment, and functional decline. The association between inflammatory signatures and plasma GFAP, but not amyloid or tau biomarkers, suggests a relationship between peripheral inflammatory profiles and glial-related biomarker alterations that requires further longitudinal investigation.

## Introduction

Alzheimer’s disease (AD) is the most common cause of dementia and represents a major global health challenge in aging populations (1–5). Despite substantial advances in understanding AD pathobiology and the emergence of disease-modifying therapies, early identification and biological characterization of individuals across different disease stages remain challenging (6). The development of biomarker-based frameworks has shifted the conceptualization of AD from a purely clinical syndrome toward a biological continuum defined by progressive interactions between neuropathological changes and clinical manifestations (7–9). However, current approaches for assessing AD pathology, including amyloid-β (Aβ) positron emission tomography (PET), cerebrospinal fluid (CSF), and advanced blood-based biomarkers, remain limited by cost, accessibility, and implementation barriers, restricting their application for large-scale screening and routine clinical risk stratification (10, 11). Therefore, identifying accessible and scalable peripheral biomarkers that provide complementary biological information beyond established AD markers remains an important research priority.

Increasing evidence suggests that AD pathophysiology extends beyond amyloid and tau accumulation and involves complex interactions among neurodegeneration, vascular dysfunction, metabolic alterations, and immune dysregulation (12, 13). Chronic low-grade inflammation associated with aging, commonly referred to as inflammaging, has emerged as an important contributor to cognitive vulnerability and neurodegenerative processes (14–16). Peripheral immune alterations may influence brain aging through multiple interconnected pathways, including endothelial dysfunction, oxidative stress, blood-brain barrier disruption, metabolic dysregulation, and modulation of central neuroinflammatory responses (17–19). Importantly, neuroinflammatory alterations involving microglial activation and astrocytic responses are increasingly recognized as important components of AD biology and may occur across the AD continuum (20, 21). Recent biomarker studies have highlighted inflammatory and glial-related markers, such as plasma glial fibrillary acidic protein (GFAP), as potential indicators of neuroimmune alterations associated with AD progression (22–24).

While molecular biomarkers have substantially improved AD characterization, systemic inflammatory states represent an additional biological dimension that may influence disease expression and clinical heterogeneity (25, 26). Routine hematological parameters provide an attractive approach for evaluating peripheral immune status because they are inexpensive, widely available, and readily applicable in clinical settings (27–29). Composite hematological inflammatory indices integrate multiple cellular components involved in immune activation, inflammatory regulation, and physiological resilience, potentially providing more robust measures than individual blood cell parameters (30). Among these indices, the neutrophil-to-lymphocyte ratio (NLR), systemic immune-inflammation index (SII), and hemoglobin-to-red blood cell distribution width ratio (HRR) capture complementary aspects of systemic inflammatory and hematological alterations.

NLR reflects the balance between innate immune activation and adaptive immune regulation and has been associated with cognitive impairment and dementia-related outcomes in previous studies (26, 28, 31, 32). SII incorporates neutrophil, lymphocyte, and platelet counts and may provide a broader representation of systemic immune-inflammatory burden by integrating multiple inflammatory components (26, 33–35). HRR represents the relationship between erythrocyte homeostasis and red blood cell heterogeneity, incorporating information related to oxygen-carrying capacity, inflammation, oxidative stress, and biological aging (36). Although previous studies have suggested associations between these indices and cognitive decline, most investigations have focused on individual inflammatory markers or clinically diagnosed dementia, and whether different hematological inflammatory indices provide overlapping or complementary information across biologically defined AD stages remains unclear.

Several important knowledge gaps remain. First, previous studies have predominantly examined inflammatory markers in individuals with established dementia or compared AD patients with cognitively unimpaired controls. Whether peripheral inflammatory signatures change progressively across the biomarker-defined AD continuum, including cognitively unimpaired individuals, amyloid-positive mild cognitive impairment, and amyloid-positive AD dementia, remains insufficiently characterized. Second, individual inflammatory indices are biologically correlated and may capture partially overlapping aspects of systemic immune alterations. Whether integrating multiple inflammatory markers into a composite inflammatory signature can provide more comprehensive information than individual indices and improve disease-stage classification beyond established demographic, vascular, and genetic risk factors remains unknown. Third, although peripheral inflammation has been implicated in AD-related biological processes, the relationship between hematological inflammatory signatures and molecular markers of AD pathology and neuroinflammation remains poorly understood. In particular, whether peripheral inflammatory alterations are associated with astroglial activation reflected by plasma GFAP requires further investigation.

Therefore, this study aimed to comprehensively evaluate the association between routinely available hematological inflammatory signatures and biological progression across the amyloid-defined AD continuum. Using a clinically characterized cohort incorporating Aβ-PET imaging, plasma AD biomarkers, APOE genotyping, and detailed cognitive assessments, we investigated four specific questions: (1) whether NLR, SII, and HRR demonstrate stage-dependent alterations across cognitively unimpaired, amyloid-positive mild cognitive impairment, and amyloid-positive AD dementia groups; (2) whether these inflammatory indices are independently associated with more advanced AD continuum stages after adjustment for established demographic, vascular, and genetic risk factors; (3) whether a composite inflammatory signature derived from multiple indices provides additional value for AD continuum classification; and (4) whether peripheral inflammatory signatures are associated with cognitive impairment and AD-related plasma biomarkers, particularly markers reflecting neuroinflammatory activation.

By integrating peripheral inflammatory profiling with molecular biomarkers and clinical phenotyping, this study sought to determine whether routine hematological inflammatory signatures can serve as accessible complementary biomarkers for characterizing AD-related biological changes and improving scalable risk assessment across the AD continuum.

## Methods

### Study design and participants

This single-center, retrospective cross-sectional study consecutively enrolled participants who underwent comprehensive cognitive assessment, blood biomarker measurements, and Aβ-PET at the Department of Neurology, West China Hospital, Sichuan University, between June 2022 and April 2026. The study protocol was approved by the Biomedical Research Ethics Committee of West China Hospital (approval No. 2021-917) and was conducted in accordance with the Declaration of Helsinki. Written informed consent was obtained from all participants or their legally authorized representatives. We follow the STROBE reporting guidelines (37).

All participants underwent standardized neurological examinations and comprehensive cognitive and functional assessments, including the Mini-Mental State Examination (MMSE), Montreal Cognitive Assessment (MoCA), and Activities of Daily Living (ADL) scale. Clinical diagnoses were independently established by experienced neurologists based on comprehensive clinical evaluation, neuropsychological performance, functional status, and established diagnostic criteria.

To characterize participants across the Alzheimer’s disease (AD) continuum, individuals were classified according to their cognitive status and amyloid biomarker profile. This approach was consistent with the National Institute on Aging-Alzheimer’s Association (NIA-AA) research framework, which conceptualizes AD as a biological continuum characterized by the interaction between neuropathological biomarkers and clinical manifestations (7).

### Group classification

Participants were categorized into three groups representing sequential clinical stages along the Alzheimer’s disease continuum based on cognitive status and Aβ-PET status: Cognitively unimpaired (CU-Aβ^-^): Participants were classified as cognitively unimpaired with negative amyloid status (CU-Aβ^-^) if they had no objective evidence of cognitive impairment, normal performance on standardized cognitive assessments, preserved functional abilities, and negative Aβ-PET results. This group served as the reference group representing the amyloid-negative reference state of the amyloid-defined Alzheimer’s disease continuum.

Amyloid-positive mild cognitive impairment (MCI-Aβ^+^): Participants with objective cognitive impairment but preserved independence in daily activities were classified as having mild cognitive impairment. Those with abnormal Aβ-PET findings were categorized as MCI-Aβ^+^, representing the prodromal symptomatic stage within the Alzheimer’s disease continuum.

Amyloid-positive dementia (AD-Aβ^+^): Participants with objective cognitive impairment accompanied by significant impairment in daily functioning and fulfilling clinical criteria for dementia were classified as AD-Aβ^+^ if they exhibited abnormal Aβ-PET findings. This group represented the clinically manifest stage of the Alzheimer’s disease continuum.

The combination of cognitive staging and amyloid biomarker status was used to capture disease progression from cognitively unimpaired individuals to symptomatic amyloid-positive cognitive impairment and dementia stages, consistent with current biomarker-based frameworks for AD research.

Participants were eligible for inclusion if they met all of the following criteria: (1) Completed standardized clinical evaluation, including demographic assessment, neurological examination, cognitive testing, functional assessment, blood biomarker measurement, and Aβ-PET imaging. (2) Were classified into one of the three predefined Alzheimer’s disease continuum groups: CU-Aβ^-^, MCI-Aβ^+^, or AD-Aβ^+^. (3) Had complete and reliable clinical information, including demographic characteristics, vascular and metabolic risk factors, APOE genotyping, cognitive assessments, laboratory measurements, and imaging data. (4) Were able to complete cognitive assessments and peripheral blood collection according to the study protocol.

Participants were excluded if they had: (1) a history of stroke, transient ischemic attack, or other cerebrovascular diseases; (2) major neurological disorders, including traumatic brain injury, brain tumors, epilepsy, Parkinson’s disease, or other neurodegenerative disorders; (3) major psychiatric disorders; (4) acute infection or systemic inflammatory diseases that could affect peripheral inflammatory indices; (5) autoimmune diseases, hematological disorders, active malignancy, severe hepatic or renal dysfunction, or other systemic diseases that could influence inflammatory status; (6) incomplete demographic, cognitive, laboratory, genetic, or imaging information; or (7) inability to complete cognitive assessments or blood sampling.

### Data collection and variable definitions

Demographic and clinical characteristics were collected through standardized clinical assessments, including age, sex, education level, body mass index (BMI), and vascular risk factors, including hypertension, diabetes mellitus, and hyperlipidemia.

Venous blood samples were collected by trained nurses in the Department of Neurology, West China Hospital, Sichuan University, in the early morning after overnight fasting (6–8 hours). Complete blood count (CBC) parameters were automatically measured using an XN-9100 hematology analyzer (Sysmex Corporation, Japan) with corresponding reagents. Hematological parameters included erythrocyte-related indices (red blood cell count, hemoglobin concentration, hematocrit, mean corpuscular volume, mean corpuscular hemoglobin, mean corpuscular hemoglobin concentration, and red cell distribution width), leukocyte-related indices (white blood cell count, neutrophil count, and lymphocyte count), and platelet-related indices (platelet count).

Based on routine hematological parameters, inflammation-related indices were calculated as follows: neutrophil-to-lymphocyte ratio (NLR) was calculated as neutrophil count divided by lymphocyte count; systemic immune-inflammation index (SII) was calculated as platelet count multiplied by neutrophil count divided by lymphocyte count; platelet-to-lymphocyte ratio (PLR) was calculated as platelet count divided by lymphocyte count; and hemoglobin-to-red cell distribution width ratio (HRR) was calculated as hemoglobin concentration divided by red cell distribution width.

APOE genotyping was performed as part of routine clinical assessment using a polymerase chain reaction (PCR)-based method. Genomic DNA was isolated from peripheral blood cells, and APOE genotypes were determined using a Human APOE ϵ2/ϵ3/ϵ4 Genotyping Kit (Memorigen, Xiamen, China) on an ABI 7500 FAST real-time PCR system (Applied Biosystems, Thermo Fisher Scientific, Waltham, USA). The assay specifically targeted the two APOE-defining single nucleotide polymorphisms (SNPs), rs429358 and rs7412, which were used to determine APOE ϵ2, ϵ3, and ϵ4 alleles according to the manufacturer’s protocol. All procedures were performed strictly according to the manufacturer’s instructions (38), and the detailed methodology has been described previously. APOE ϵ4 carrier status was defined as the presence of at least one ϵ4 allele.

Amyloid-β (Aβ) pathology was assessed using positron emission tomography (PET) imaging with [18F]florbetapir. PET acquisition and processing procedures have been described previously. Briefly, PET images were analyzed using standardized uptake value ratio (SUVR)-based methods, and Aβ positivity was determined according to previously validated SUVR thresholds.

### AD blood biomarkers

Peripheral blood samples were collected following an overnight fast into EDTA-treated tubes and processed within 30 minutes of collection. Plasma was separated by centrifugation at 845 × g for 15 minutes at 4 °C, subsequently aliquoted, and stored at −80 °C until biomarker measurement. Plasma levels of Alzheimer’s disease-related biomarkers, including Aβ40, Aβ42, p-tau181, and p-tau217, as well as neurodegeneration and astroglial activation markers (NfL and GFAP), were quantified using ultrasensitive single-molecule array (Simoa) immunoassays on the Quanterix platform. All assays were performed in a single analytical batch by investigators blinded to participants’ clinical characteristics. The measurements were conducted in accordance with the manufacturer’s instructions, and calibration standards and internal quality controls were included in each assay run to ensure assay accuracy and reproducibility.

### Construction of a composite inflammatory index

To capture the shared inflammatory burden reflected by multiple correlated hematological inflammatory indices, a composite inflammatory index was derived using principal component analysis (PCA) (39).Three inflammation-related indices, including the neutrophil-to-lymphocyte ratio (NLR), systemic immune-inflammation index (SII), and hemoglobin-to-red blood cell distribution width ratio (HRR), were included in the PCA. Prior to PCA, all inflammatory markers were transformed into standardized z-scores to ensure comparability across different measurement scales. Because higher HRR values have been inversely associated with systemic inflammatory status compared with NLR and SII, HRR was directionally aligned before analysis by multiplying its standardized value by -1. PCA was subsequently performed on the three standardized inflammatory indicators, and the first principal component (PC1), which explained the largest proportion of the total variance, was extracted as the composite inflammatory index (Inflam_PC1). Higher Inflam_PC1 values indicated a greater overall inflammatory burden. The use of a PCA-derived inflammatory index enabled integration of correlated inflammatory markers into a single continuous measure, providing a more comprehensive representation of systemic inflammatory status than individual biomarkers alone.

### Statistical analysis

Continuous variables were assessed for distributional characteristics using the Shapiro-Wilk test and were summarized as mean ± standard deviation (SD) or median (interquartile range [IQR]), as appropriate. Homogeneity of variance was evaluated before performing parametric analyses. Comparisons of continuous variables among groups were conducted using one-way analysis of variance (ANOVA) with Tukey’s *post hoc* test for normally distributed variables with equal variances, or the Kruskal-Wallis test followed by Dunn’s *post hoc* comparisons with Holm adjustment for non-normally distributed variables. Categorical variables were presented as frequencies and percentages and compared using the χ² test or Fisher’s exact test, as appropriate.

Associations between variables were evaluated using Spearman rank correlation analyses. Multiple comparisons in correlation analyses were controlled using the Benjamini-Hochberg false discovery rate (FDR) procedure.

Given that cognitive status and available amyloid biomarker profiles represented an ordered AD continuum, participants were categorized into three sequential stages: cognitively unimpaired (CU-Aβ^-^), amyloid-positive mild cognitive impairment (MCI-Aβ^+^), and amyloid-positive Alzheimer’s disease dementia (AD-Aβ^+^). A proportional-odds ordinal logistic regression model was used to evaluate the associations between hematological inflammatory indices and the probability of being classified into a more advanced stage of the AD continuum. The proportional odds assumption was formally assessed using the Brant test.

Six sequential models were constructed to evaluate the independent associations and incremental contribution of inflammatory markers. Model 1 (the base model) included established demographic, vascular, metabolic, and genetic risk factors, including age, sex, body mass index (BMI), years of education, hypertension, diabetes mellitus, hyperlipidemia, smoking status, alcohol consumption, and APOE ϵ4 carrier status. Subsequently, individual inflammatory indices were introduced into the base model: NLR (Model 2), SII (Model 3), HRR (Model 4), all three inflammatory indices simultaneously (Model 5), and the PCA-derived inflammatory component (Inflam_PC1) (Model 6). Inflam_PC1 was derived before regression modelling and was treated as an exposure variable rather than a predictive feature.

Results from ordinal logistic regression models were reported as odds ratios (ORs) with corresponding 95% confidence intervals (CIs), representing the odds of being classified into a higher stage of the AD continuum. Model performance and incremental value were assessed using likelihood ratio tests (LRTs), Akaike information criterion (AIC) comparisons, and Nagelkerke pseudo-R². The discriminatory performance of each model was evaluated using ROC curve analyses based on cumulative thresholds. Pairwise comparisons of area under the curve (AUC) values were performed using DeLong’s test, and ten-fold cross-validation was used for internal assessment of model discrimination. Inflam_PC1 was derived *a priori* before regression modeling and was treated as an exposure variable rather than a model-derived predictive feature; therefore, it was not optimized or recalculated during cross-validation.

Model calibration was assessed for the ordinal logistic regression models using the two cumulative thresholds (≥MCI-Aβ^+^ vs CU-Aβ^-^ and ≥AD-Aβ^+^ vs CU-Aβ^-^ +MCI-Aβ^+^). Calibration performance was evaluated using Brier scores, calibration intercepts, calibration slopes, and the Hosmer-Lemeshow goodness-of-fit test.

Prespecified subgroup analyses were conducted according to APOE ϵ4 carrier status, sex, and age group (<65 years versus ≥65 years). Within each subgroup, proportional-odds ordinal logistic regression models were independently fitted to evaluate the consistency of associations between inflammatory indices and AD continuum stage. Potential effect modification was examined by introducing multiplicative interaction terms between inflammatory indices and subgroup variables.

All statistical analyses were performed using R software (version 4.6.1). All tests were two-sided, and a *P* value <0.05 was considered statistically significant.

## Results

### Participant characteristics

A total of 267 participants were included in the final analysis, comprising 86 cognitively unimpaired (CU-Aβ^-^) individuals, 69 participants with amyloid-positive mild cognitive impairment (MCI-Aβ^+^), and 112 participants with amyloid-positive Alzheimer’s disease dementia (AD-Aβ^+^). Participants were categorized into three clinical and biomarker-informed stages representing increasing disease severity (**Figure 1**).

**Figure 1:**
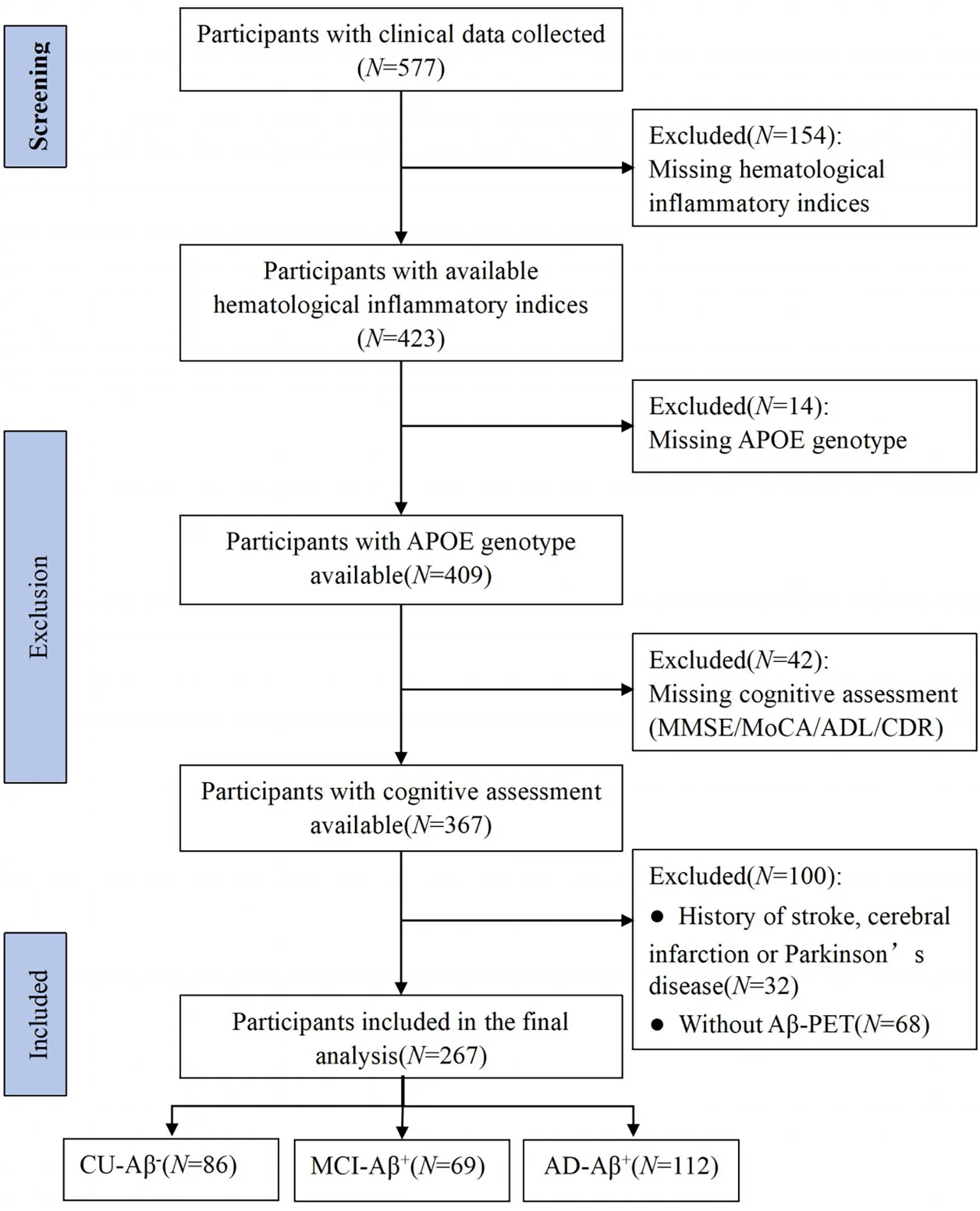
Flowchart of participant selection. Among 577 participants screened, 267 were included in the final analysis: 86 CU-Aβ−, 69 MCI-Aβ+, and 112 AD-Aβ+ participants.

Demographic and clinical characteristics differed across disease stages (**Table 1**). Participants in more advanced stages were older, had fewer years of education, and showed a higher prevalence of APOE ϵ4 carriage (all *P* < 0.05). Cognitive and functional measures demonstrated progressive impairment across the three clinical stages, with lower MMSE scores and higher ADL scores observed in MCI-Aβ^+^ and AD-Aβ^+^ groups compared with CU-Aβ^-^ individuals (both *P* < 0.001). Other demographic and vascular risk factors were comparable among groups.

**Table 1:** Baseline characteristics of participants across the Alzheimer’s disease continuum.

Variable	CU-Aβ^-^ (N=86)	MCI-Aβ^+^ (N=69)	AD-Aβ^+^ (N=112)	Overall_*P*	*P*1	*P*2	*P*3
Sex	86 (100%)	69 (100%)	112 (100%)	0.267	0.751	0.503	0.471
Female	45 (52.3%)	34 (49.3%)	68 (60.7%)				
Male	41 (47.7%)	35 (50.7%)	44 (39.3%)				
Age	60 (55-66)	73 (62-78)	71.5 (65.75-77)	<0.001***	<0.001***	<0.001***	0.949
BMI	22.22 (20.51-24.33)	20.97 (19.62-23.31)	21.47 (18.57-23.44)	0.028*	0.062	0.042*	0.983
Education	15 (12-16)	12 (9-15)	9 (5-12)	<0.001***	<0.001***	<0.001***	<0.001***
Hypertension	86 (100%)	69 (100%)	112 (100%)	0.720	1.000	1.000	1.000
0	64 (74.4%)	48 (69.6%)	78 (69.6%)				
1	22 (25.6%)	21 (30.4%)	34 (30.4%)				
Diabetes	86 (100%)	69 (100%)	112 (100%)	0.077	0.175	1.000	0.175
0	74 (86%)	51 (73.9%)	96 (85.7%)				
1	12 (14%)	18 (26.1%)	16 (14.3%)				
Hyperlipidemia	86 (100%)	69 (100%)	112 (100%)	0.066	0.690	0.186	0.097
0	65 (75.6%)	50 (72.5%)	96 (85.7%)				
1	21 (24.4%)	19 (27.5%)	16 (14.3%)				
Smoking	86 (100%)	69 (100%)	112 (100%)	0.903	1.000	1.000	1.000
0	71 (82.6%)	55 (79.7%)	91 (81.2%)				
1	15 (17.4%)	14 (20.3%)	21 (18.8%)				
Alcohol	86 (100%)	69 (100%)	112 (100%)	0.734	1.000	1.000	1.000
0	69 (80.2%)	58 (84.1%)	89 (79.5%)				
1	17 (19.8%)	11 (15.9%)	23 (20.5%)				
APOE ϵ4	86 (100%)	69 (100%)	112 (100%)	0.010**	0.371	0.010*	0.371
Non-carrier	51 (59.3%)	33 (47.8%)	42 (37.5%)				
Carrier	35 (40.7%)	36 (52.2%)	70 (62.5%)				
MMSE	29 (28-30)	23 (21-25)	14 (10-17.25)	<0.001***	<0.001***	<0.001***	<0.001***
ADL	14 (14-14)	14 (14-16)	20 (16-30)	<0.001***	<0.001***	<0.001***	<0.001***

CU, cognitively unimpaired; MCI, Mild Cognitive Impairment; AD, Alzheimer’s disease; BMI, Body Mass Index; MoCA, Montreal Cognitive Assessment; MMSE, Mini-Mental State Examination; ADL, Activities of Daily Living. *P*1, CU-Aβ^-^ *vs* MCI-Aβ^+^; *P*2, CU-Aβ^-^ *vs* AD-Aβ^+^; *P*3, MCI-Aβ^+^ *vs* AD-Aβ^+^. *P < 0.05; ***P < 0.001.

### Peripheral inflammatory signatures across the Alzheimer’s disease continuum

Peripheral inflammatory indices demonstrated progressive alterations across the Alzheimer’s disease continuum (**Table 2**; **Figure 2**). NLR levels increased across disease stages from CU-Aβ^-^ to MCI-Aβ^+^ and AD-Aβ^+^ groups (median, 1.83, 2.11, and 2.30, respectively; *P* = 0.005), with significantly higher levels in AD-Aβ^+^ compared with CU-Aβ^-^ individuals (*P* = 0.003). Similarly, SII levels increased across disease stages and were elevated in AD-Aβ^+^ compared with CU-Aβ^-^ participants (*P* = 0.049). In contrast, HRR showed a progressive decrease across the continuum (*P* = 0.031), with lower levels observed in AD-Aβ^+^ than CU-Aβ^-^ individuals (*P* = 0.028). Other routine hematological parameters showed limited differences among groups. Although RBC and RDW-SD differed significantly, conventional blood cell indices, including hemoglobin, hematocrit, platelet count, leukocyte count, neutrophil count, and lymphocyte count, were comparable across groups (all *P* > 0.05).

**Table 2:** Altered peripheral inflammatory signatures across the Alzheimer’s disease continuum.

Variable	CU-Aβ^-^ (N=86)	MCI-Aβ^+^ (N=69)	AD-Aβ^+^ (N=112)	Overall_*P*	*P*1	*P*2	*P*3
NLR	1.83 (1.4-2.38)	2.11 (1.66-2.8)	2.3 (1.65-3.22)	0.005**	0.120	0.003**	0.281
HRR	10.85 (10-11.68)	10.65 (9.86-11.14)	10.23 (9.51-11.27)	0.031*	0.194	0.028*	0.494
SII	366.41 (273.67-507.44)	354.42 (274.34-478.41)	419.19 (314.06-601.97)	0.026*	0.886	0.049*	0.072
PLR	113.37 (93.17-141.53)	108.7 (84.07-140.15)	128.23 (94.99-159.03)	0.061	0.453	0.237	0.072
RBC	4.58 (4.31-4.85)	4.44 (4.17-4.66)	4.42 (4.11-4.76)	0.022*	0.045*	0.045*	0.846
HGB	137 (129-149.75)	137 (130-143)	135 (125-145)	0.210	0.786	0.231	0.786
Hct	0.42 (0.4-0.45)	0.42 (0.4-0.44)	0.41 (0.39-0.45)	0.327	0.827	0.406	0.827
MCV	93.35 (91.05-95.95)	94.7 (92.3-97)	94.3 (90.88-96.82)	0.087	0.081	0.438	0.438
MCH	30.65 (29.22-31.5)	31 (29.8-32)	30.4 (29.4-31.4)	0.175	0.255	0.810	0.255
MCHC	325.5 (320-332)	326 (321-331)	324.5 (317-331.25)	0.574	1.000	1.000	1.000
RDW-SD	43.65 (41.32-45.9)	44.8 (43.1-46.1)	44.9 (42.7-46.95)	0.012*	0.037*	0.017*	0.914
RDW-CV	12.75 (12.4-13.3)	12.9 (12.5-13.4)	13.1 (12.57-13.6)	0.124	0.587	0.123	0.587
PLT	195 (169.25-223.5)	180 (152-213)	192 (156.75-240.25)	0.053	0.084	0.828	0.084
WBC	5.64 (4.76-6.54)	5.5 (4.96-6.58)	5.7 (4.8-7.21)	0.580	0.977	0.959	0.977
Neutrophil	3.15 (2.54-3.97)	3.31 (2.88-4.15)	3.45 (2.77-4.44)	0.100	0.260	0.113	0.729
Lymphocyte	1.71 (1.39-2.09)	1.56 (1.3-1.84)	1.55 (1.22-1.95)	0.108	0.322	0.116	0.649

NLR, neutrophil-to-lymphocyte ratio; HRR, hemoglobin-to-red cell distribution width ratio; SII, systemic immune-inflammation index; PLR, platelet-to-lymphocyte ratio. CU, cognitively unimpaired; MCI, Mild Cognitive Impairment; AD, Alzheimer’s disease.

*P*1, CU-Aβ^-^ *vs* MCI-Aβ^+^; *P*2, CU-Aβ^-^ *vs* AD-Aβ^+^; *P*3, MCI-Aβ^+^ *vs* AD-Aβ^+^. *P < 0.05; **P < 0.01.

**Figure 2:**
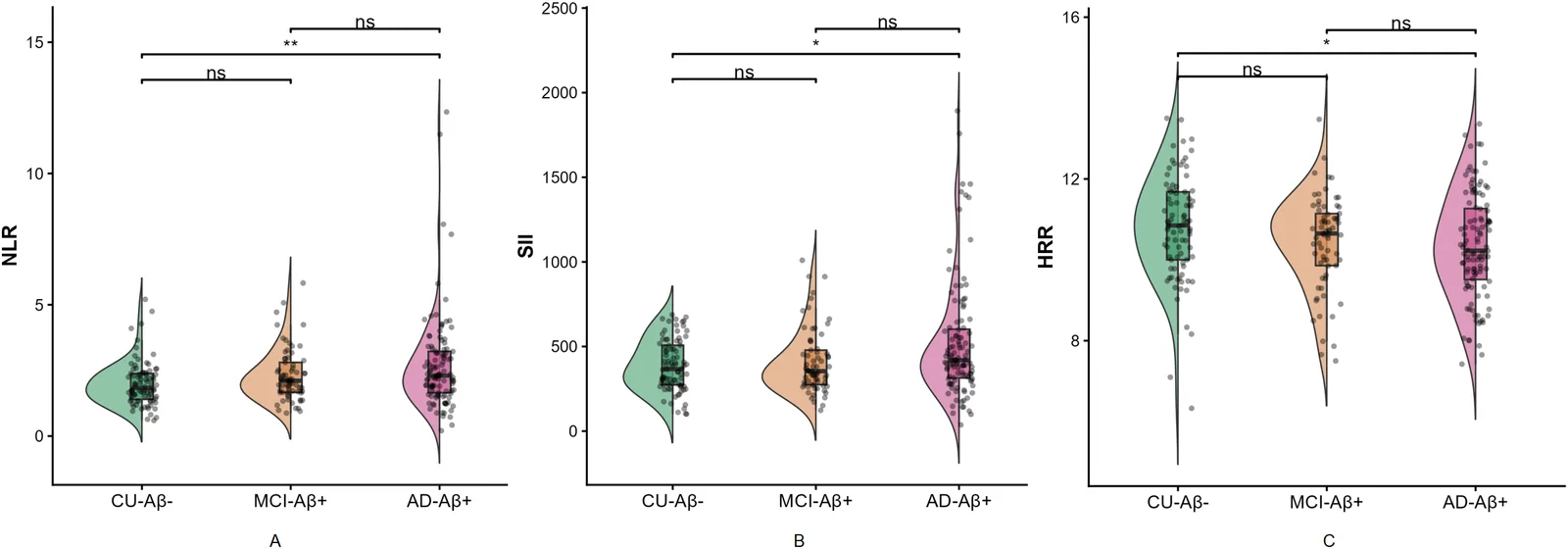
Distributions of NLR, SII, and HRR across the CU-Aβ−, MCI-Aβ+, and AD-Aβ+ groups. Violin plots with overlaid box plots and individual data points show the distributions of **(A)** NLR, **(B)** SII, and **(C)** HRR. ns, not significant; *P<0.05; **P<0.01.

### Plasma Alzheimer’s disease biomarkers across the Alzheimer’s disease continuum

Plasma AD biomarkers demonstrated expected stage-related differences across the Alzheimer’s disease continuum (**Table 3**; **Figure 3**). Compared with CU-Aβ^-^ individuals, MCI-Aβ^+^ and AD-Aβ^+^ participants showed higher plasma Aβ42/Aβ40 ratios and increased concentrations of p-tau181 and p-tau217 (all *P* < 0.001). Plasma GFAP concentrations increased progressively across disease stages (median, 9.05, 12.39, and 18.14, respectively; *P* < 0.001), whereas plasma NfL levels were higher among amyloid-positive individuals than CU-Aβ^-^ participants (*P* = 0.015).

**Table 3:** Plasma Alzheimer’s disease biomarkers across the Alzheimer’s disease continuum.

Variable	CU-Aβ^-^ (N=41)	MCI-Aβ^+^ (N=45)	AD-Aβ^+^ (N=53)	Overall_*P*	*P*1	*P*2	*P*3
Aβ42	39.82 (24.69-53.84)	68.36 (47.68-86.41)	76.28 (55.79-99.55)	<0.001***	<0.001***	<0.001***	0.2311
Aβ40	441.68 (275.39-519.28)	427.68 (361.85-504.98)	399.72 (265.54-489.64)	0.7829			
Aβ42/40	0.09 (0.06-0.19)	0.15 (0.11-0.22)	0.18 (0.12-0.29)	<0.001***	0.02*	<0.001***	0.121
pTau-181	2.07 (1.12-4.67)	4.71 (2.14-8.16)	5.43 (3.68-9.91)	<0.001***	0.02*	<0.001***	0.051
pTau-217	3.02 (1.75-6.36)	9.37 (3.36-14.22)	10.12 (6.25-17.13)	<0.001***	0.0013**	<0.001***	0.113
NFL	8.49 (4.39-16.19)	15.15 (7.49-22.74)	14.24 (9.35-28.96)	0.015*	0.1973	0.0113*	0.2246
GFAP	9.05 (6.3-11.77)	12.39 (9.21-16.62)	18.14 (13.3-23.56)	<0.001***	<0.001***	<0.001***	<0.001***
Aβ42	39.82 (24.69-53.84)	68.36 (47.68-86.41)	76.28 (55.79-99.55)	<0.001***	<0.001***	<0.001***	0.2311

*P*1, CU-Aβ^-^ *vs* MCI-Aβ^+^; *P*2, CU-Aβ^-^ *vs* AD-Aβ^+^; *P*3, MCI-Aβ^+^ *vs* AD-Aβ^+^.*P < 0.05; **P < 0.01; ***P < 0.001.

CU, cognitively unimpaired; MCI, Mild Cognitive Impairment; AD, Alzheimer’s disease.

**Figure 3:**
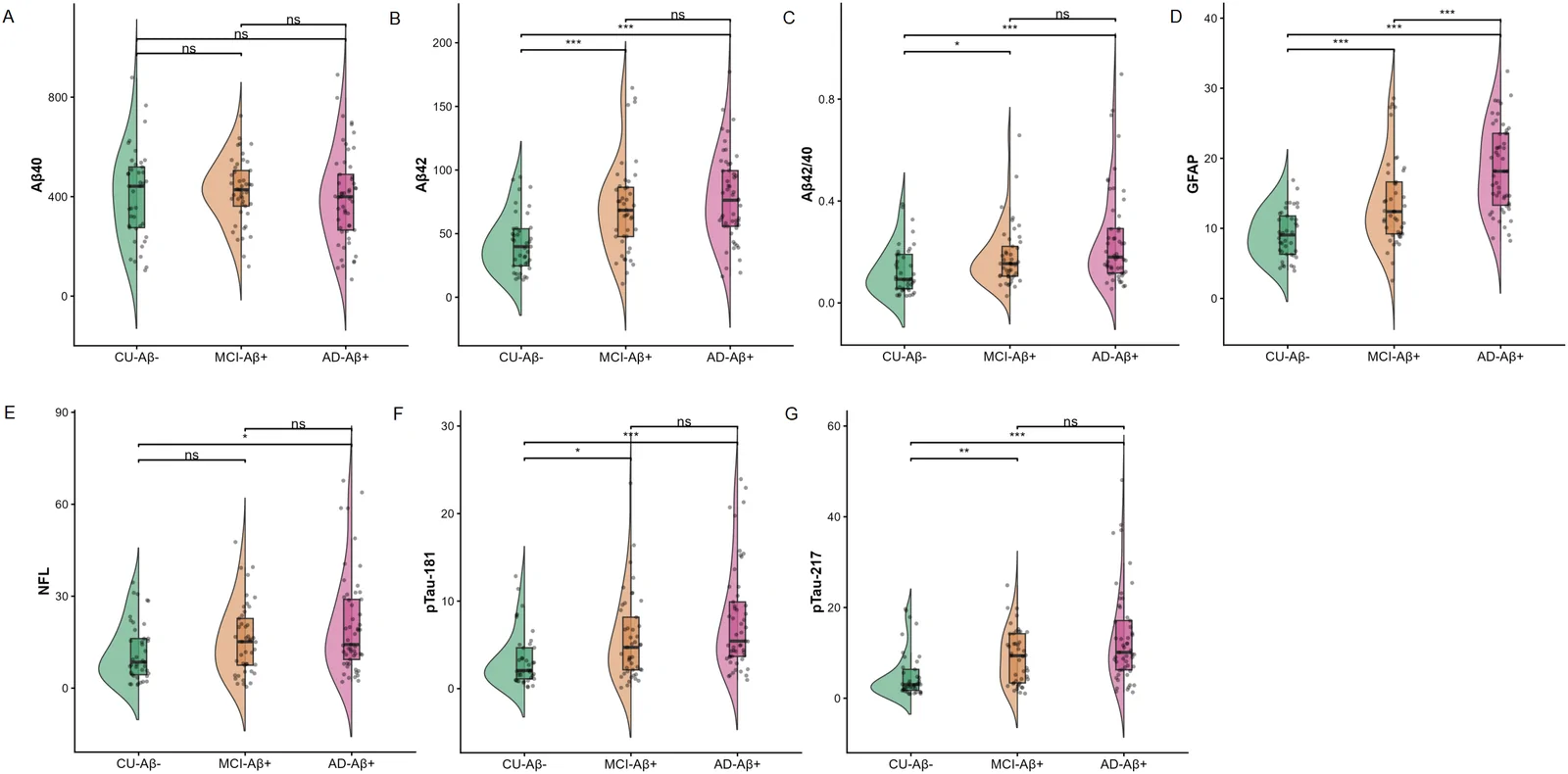
Violin plots **(A–G)** show the distributions of Aβ40, Aβ42, Aβ42/40, GFAP, NfL, p-tau181, and p-tau217 across the CU-Aβ−, MCI-Aβ+, and AD-Aβ+ groups. Group differences are indicated as ns, *P < 0.05, **P < 0.01, and ***P < 0.001.

### Peripheral inflammatory signatures independently associate with Alzheimer’s disease continuum stage

The proportional odds assumption was satisfied for the primary inflammatory predictors based on the Brant test, supporting the appropriateness of ordinal logistic regression analyses (**Supplementary Table 1**). Ordinal logistic regression models were used to evaluate whether inflammatory signatures were independently associated with more advanced Alzheimer’s disease continuum stages (**Table 4**). After adjustment for demographic, vascular, metabolic, and genetic factors, higher NLR and SII levels remained independently associated with advanced disease stages. Each 1-unit increase in NLR was associated with higher odds of being classified into a more advanced stage (OR = 1.54, 95% CI: 1.25-1.91, *P* < 0.001), whereas each 100-unit increase in SII was associated with a 29% higher odds (OR = 1.29, 95% CI: 1.15-1.45, *P* < 0.001). HRR was not independently associated with disease stage (OR = 0.94, 95% CI: 0.76-1.16, *P* = 0.571). Inflam_PC1 explained 60.5% of the variance among inflammatory indices, with dominant loadings from NLR and SII, reflecting the shared inflammatory pattern captured by these correlated indices. Inflam_PC1 was also significantly association with disease stage (OR = 1.63 per SD increase, 95% CI:1.30-2.03, *P* < 0.001; **Supplementary Table 2**). Age, education, and APOE ϵ4 status remained independently associated with disease stage across models.

**Table 4:** Multivariable ordinal logistic regression analysis of inflammatory signatures across Alzheimer’s disease continuum stages.

Variable	Model 1 (base)	Model 2 (NLR)	Model 3 (SII)	Model 4 (HRR)	Model 5 (all)	Model 6 (PCA)
Age (years)	1.07 (1.04–1.10)***	1.07 (1.05–1.10)***	1.07 (1.05–1.10)***	1.07 (1.04–1.10)***	1.07 (1.04–1.10)***	1.08 (1.05–1.10)***
Sex(Male)	0.84 (0.46–1.56)	0.72 (0.39–1.36)	0.86 (0.46–1.61)	0.87 (0.47–1.62)	0.83 (0.43–1.59)	0.77 (0.41–1.45)
BMI (kg/m²)	0.96 (0.89–1.04)	0.97 (0.89–1.05)	0.96 (0.88–1.04)	0.96 (0.89–1.04)	0.96 (0.89–1.05)	0.96 (0.88–1.04)
Education (years)	0.81 (0.76–0.86)***	0.81 (0.76–0.86)***	0.81 (0.76–0.87)***	0.81 (0.76–0.86)***	0.81 (0.76–0.87)***	0.81 (0.76–0.87)***
Hypertension (yes*vs* no)	0.97 (0.53–1.77)	0.92 (0.50–1.72)	0.83 (0.45–1.55)	0.96 (0.53–1.75)	0.84 (0.45–1.58)	0.88 (0.47–1.63)
Diabetes (yes *vs* no)	0.95 (0.49–1.86)	0.88 (0.44–1.76)	1.11 (0.55–2.21)	0.93 (0.47–1.82)	1.00 (0.49–2.04)	0.99 (0.50–1.98)
Hyperlipidemia (yes *vs* no)	0.70 (0.37–1.31)	0.61 (0.32–1.16)	0.55 (0.29–1.05)	0.69 (0.37–1.29)	0.55 (0.28–1.06)	0.58 (0.30–1.10)
Smoking (yes *vs* no)	1.26 (0.56–2.83)	1.14 (0.50–2.60)	1.12 (0.49–2.55)	1.27 (0.56–2.85)	1.13 (0.49–2.57)	1.12 (0.49–2.55)
Alcohol (yes *vs* no)	1.36 (0.60–3.07)	1.39 (0.61–3.19)	1.43 (0.62–3.26)	1.37 (0.61–3.08)	1.42 (0.62–3.27)	1.40 (0.61–3.22)
APOE ϵ4	1.93 (1.17–3.19)*	2.19 (1.30–3.66)**	2.37 (1.40–4.00)**	1.91 (1.15–3.16)*	2.31 (1.36–3.90)**	2.31 (1.37–3.89)**
NLR	NA	1.54 (1.25–1.91)***	NA	NA	1.19 (0.83–1.70)	NA
SII (per 100 units)	NA	NA	1.29 (1.15–1.45)***	NA	1.19 (0.97–1.46)	NA
HRR	NA	NA	NA	0.94 (0.76–1.16)	0.93 (0.75–1.16)	NA
PCA (per 1 SD)	NA	NA	NA	NA	NA	1.63 (1.30–2.03)***

Data are presented as odds ratio (95% confidence interval). All models adjusted for age, sex, BMI, education, hypertension, diabetes, hyperlipidemia, smoking, alcohol, and APOE ϵ4 dosage. PCA,Principal Component Analysis.

*P < 0.05; **P < 0.01; ***P < 0.001; NA, variable not included in the model. Bold values indicate statistical significance (P < 0.05).

### Incremental value of inflammatory signatures for Alzheimer’s disease continuum classification

Adding inflammatory signatures improved model performance compared with the base model. All inflammatory models showed lower AIC values and higher Nagelkerke pseudo-R² values than the base model. The model incorporating all inflammatory indices achieved the highest Nagelkerke pseudo-R² (0.442), whereas the Inflam_PC1 model showed the lowest AIC (471.007), indicating improved model fit after incorporating inflammatory signatures (**Supplementary Table 3**). ROC analyses demonstrated moderate discrimination between CU-Aβ^-^ and AD-Aβ^+^ groups (AUC, 0.78-0.85), whereas discrimination between adjacent disease stages remained modest (AUC, 0.62-0.70), indicating limited ability of inflammatory signatures to distinguish neighboring stages within the Alzheimer’s disease continuum (**Supplementary Figure 1**). Calibration analyses were performed for the two cumulative thresholds of the ordinal logistic regression models. Although Brier scores indicated relatively low overall prediction error and Hosmer-Lemeshow tests applied to the cumulative binary thresholds, calibration intercepts and slopes deviated from their ideal values, suggesting that calibration performance should be interpreted cautiously (**Supplementary Table 4**).

### Associations between inflammatory signatures and cognitive and functional measures

Associations of peripheral inflammatory indices with cognitive and functional measures were examined across disease stages (**Figure 4**; **Supplementary Table 5**). Higher SII and NLR levels were associated with poorer cognitive performance and greater functional impairment, whereas higher HRR was associated with better cognitive and functional measures. The strength of associations varied across disease stages, with the most pronounced relationship between SII and cognition observed in the MCI-Aβ^+^ group (ρ = -0.329, *P* = 0.007).

**Figure 4:**
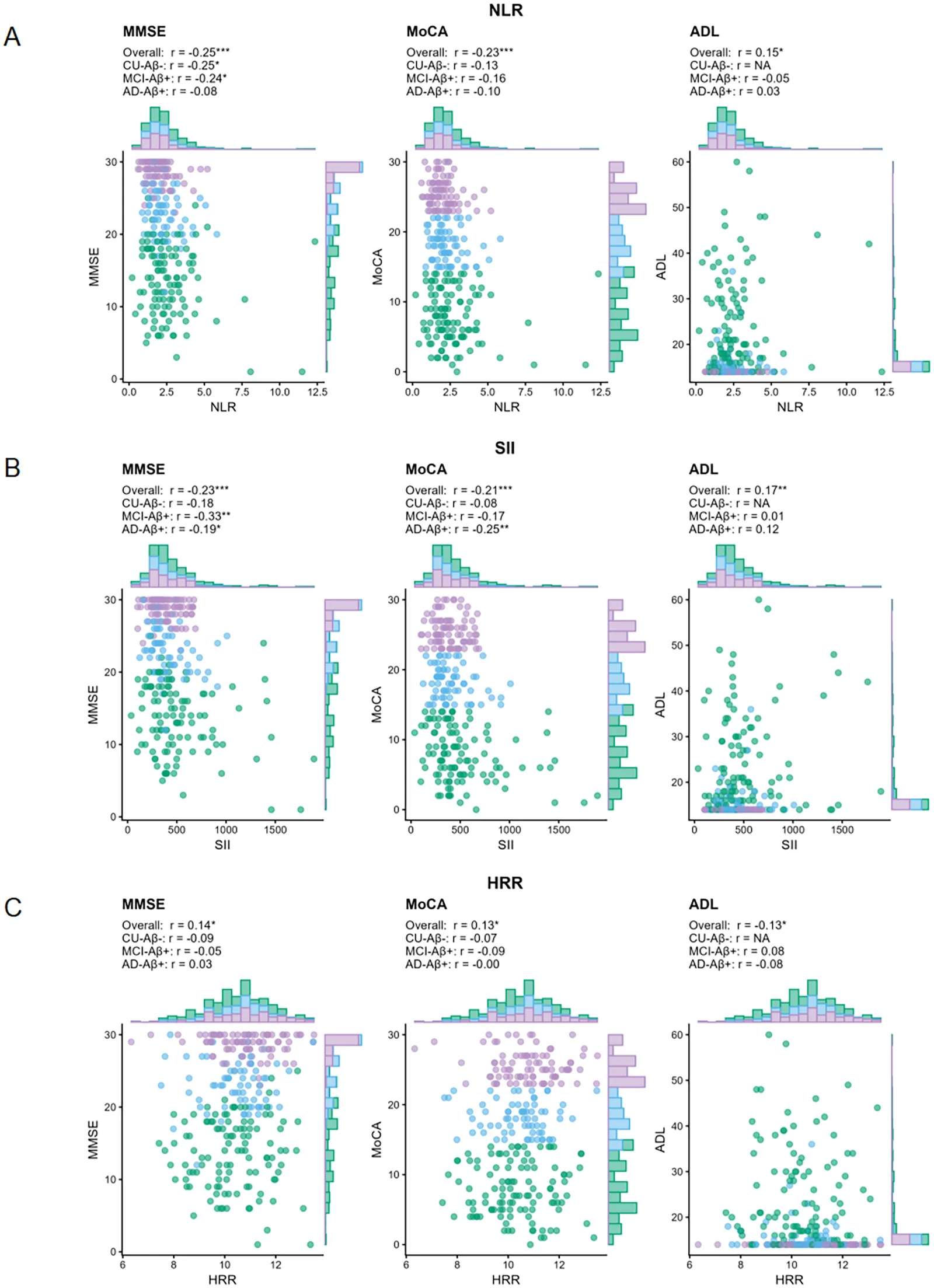
Scatterplot matrix **(A–C)** showing the associations of NLR, SII, and HRR with MMSE, MoCA, and ADL scores. Correlation coefficients are shown for each group and for the overall sample, with marginal histograms displaying the distributions of the variables.

### Robustness of inflammatory associations across demographic and genetic subgroups

Stratified analyses demonstrated that associations of NLR and SII with advanced Alzheimer’s disease continuum stages remained consistent across sex, age groups, and APOE ϵ4 carrier status (**Supplementary Tables 6**, **7**). No significant interactions were observed between inflammatory signatures and sex, age, or APOE ϵ4 status after FDR correction (all FDR-adjusted *P* > 0.40).

### Exploratory associations between inflammatory signatures and plasma Alzheimer’s disease biomarkers

Exploratory correlation analyses were performed among participants with available plasma biomarker measurements (n = 139; CU-Aβ^-^, n = 41; MCI-Aβ^+^, n = 45; AD-Aβ^+^, n = 53) to investigate associations between peripheral inflammatory indices and plasma AD biomarkers (**Figure 5**; **Supplementary Figure 2**). In the overall cohort, higher NLR and SII levels were positively associated with plasma GFAP concentrations (NLR: ρ = 0.261, FDR-adjusted *P* = 0.032; SII: ρ = 0.236, FDR-adjusted *P* = 0.037), whereas HRR showed an inverse association with GFAP levels (ρ = −0.250, FDR-adjusted *P* = 0.032). Among AD-Aβ^+^ participants, NLR showed a stronger positive association with plasma GFAP concentrations (ρ = 0.478, FDR-adjusted *P* = 0.007). After correction for multiple comparisons, no significant correlations were observed between inflammatory indices and plasma amyloid or tau biomarkers.

**Figure 5:**
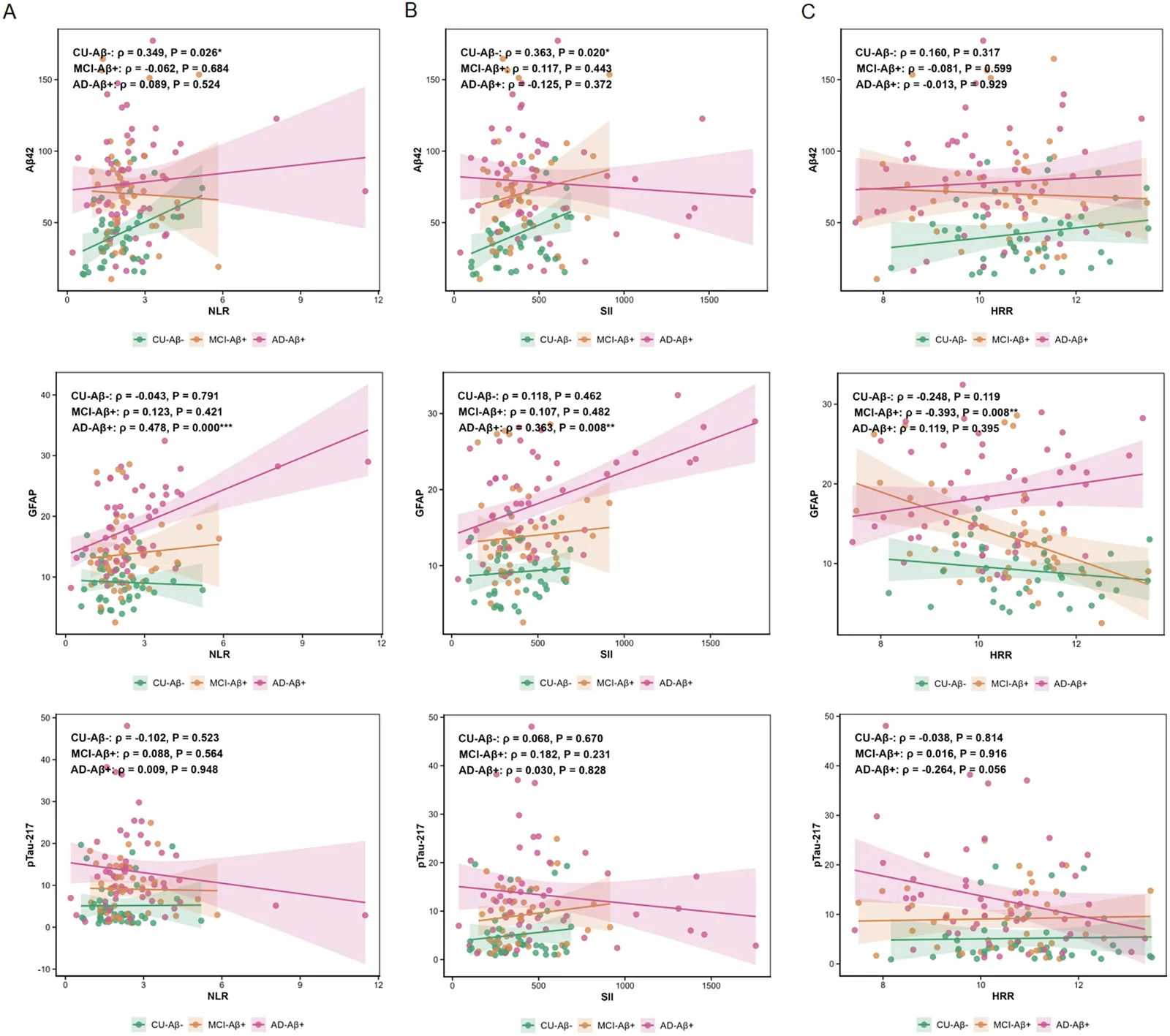
Scatterplots show the associations of Aβ42, GFAP, and p-tau217 with NLR, SII, and HRR across the CU-Aβ−, MCI-Aβ+, and AD-Aβ+ groups. Regression lines with 95% confidence intervals, correlation coefficients, and P values are shown for each group.

## Discussion

In this cross-sectional study, we investigated whether routinely available peripheral inflammatory signatures were associated with biological and clinical stages across the Alzheimer’s disease (AD) continuum. By integrating hematological inflammatory indices, plasma AD biomarkers, APOE genotyping, and cognitive assessments, we demonstrated three main findings. First, peripheral inflammatory profiles showed stage-dependent alterations across the AD continuum, characterized by increased NLR and SII and decreased HRR levels in more advanced disease stages. Second, inflammatory signatures, particularly the PCA-derived composite signature (Inflam_PC1), were independently associated with more advanced AD continuum stages after adjustment for demographic, vascular, and genetic factors and their inclusion was associated with improved relative model fit beyond established risk factors. Third, inflammatory signatures were associated with cognitive and functional impairment and correlated with plasma GFAP concentrations, suggesting an association between peripheral immune alterations and selected AD-related biological features.

Increasing evidence indicates that immune dysregulation and neuroinflammation are closely involved in AD pathophysiology (12, 27, 40–42). However, currently established AD biomarkers, including amyloid and tau biomarkers measured by PET imaging, cerebrospinal fluid, or blood-based assays, remain limited by cost, accessibility, and implementation barriers in routine clinical settings (10). Therefore, identifying inexpensive and scalable peripheral markers that capture additional biological dimensions of AD represents an important research priority. Hematological inflammatory indices derived from routine blood counts, such as NLR and SII, provide composite measures reflecting multiple immune components and have been increasingly investigated in relation to cognitive impairment and neurodegenerative diseases (43, 44).

In the present study, NLR and SII increased progressively across the AD continuum, whereas HRR demonstrated an opposite pattern. These findings are consistent with previous evidence linking peripheral inflammatory alterations with cognitive impairment and Alzheimer’s disease-related biological changes (44, 45). Importantly, our findings extends previous work by demonstrating that inflammatory alterations are detectable within an amyloid-defined AD framework, rather than being restricted to individuals who have already developed dementia. This finding suggests that peripheral inflammatory signatures are associated with more advanced Alzheimer’s stages. However, the cross-sectional design precludes causal inference, and longitudinal studies are needed to clarify the temporal dynamics.

Among the evaluated inflammatory indices, the PCA-derived composite inflammatory signature (Inflam_PC1) showed the strongest association with AD continuum stage. Unlike individual inflammatory markers, composite signatures may better capture shared biological variation across correlated immune-related parameters and reduce the influence of measurement variability from single indicators. In particular, SII integrates information from neutrophils, lymphocytes, and platelets, thereby reflecting multiple components of systemic immune activation. In our analyses, inflammatory signatures remained associated with disease stage after adjustment for APOE ϵ4 status and other established risk factors, indicating that peripheral inflammatory patterns may provide complementary information beyond conventional demographic and genetic markers. Nevertheless, these findings should be interpreted as evidence of association rather than proof that peripheral inflammation directly drives AD progression.

The relationship between peripheral inflammation and AD-related molecular pathology remains complex. In our exploratory analyses, inflammatory signatures were not significantly associated with plasma amyloid or tau biomarkers after correction for multiple comparisons, but demonstrated consistent associations with plasma GFAP. GFAP is considered a marker related to astrocytic activation and has shown associations with Aβ-related pathology and clinical progression across the AD continuum (46, 47). The selective association between peripheral inflammatory signatures and GFAP may indicate that hematological inflammatory alterations capture an immune-related biological dimension more closely linked to glial responses than to amyloid or tau burden alone. Although the cross-sectional design precludes causal inference, this pattern suggests a potentially closer connection between peripheral inflammation and astrocytic activity in the AD continuum. Previous studies have highlighted complex interactions between peripheral immune alterations and central inflammatory responses involving astrocytes, microglia, and immune signaling pathways (8, 41, 48). However, because peripheral inflammatory indices are indirect markers of systemic immune status, future studies integrating longitudinal inflammatory profiling and multimodal neuroimaging will be necessary to clarify the underlying biological mechanisms.

Beyond biological associations, inflammatory signatures demonstrated clinical relevance through their relationships with cognitive and functional impairment. Higher SII and NLR levels were associated with poorer cognitive performance and greater functional impairment, whereas higher HRR levels showed opposite associations. These findings are consistent with previous evidence linking peripheral inflammatory markers with cognitive decline and dementia severity (28, 36). However, the relatively modest effect sizes suggest that peripheral inflammation represents one component within a complex network of biological processes contributing to cognitive impairment, rather than an independent determinant of clinical severity.

We further examined whether demographic and genetic characteristics modified the association between inflammatory signatures and AD continuum stage. Associations between inflammatory indices and disease stage remained consistent across sex, age groups, and APOE ϵ4 carrier status, with no significant interaction effects detected. These findings suggest that peripheral inflammatory alterations may represent a relatively general feature associated with AD-related biological changes rather than being limited to specific demographic or genetic subgroups. Given that APOE influences both amyloid metabolism and immune regulation, longitudinal studies will be important to determine whether genetic background modifies the temporal relationship between systemic inflammation and AD pathology.

This study has several strengths. We evaluated multiple routinely available inflammatory indices within an amyloid-defined clinical AD continuum framework rather than comparing only dementia and cognitively unimpaired groups. The integration of inflammatory signatures with plasma AD biomarkers, APOE genotype, and detailed cognitive assessments allowed a multidimensional evaluation of peripheral inflammation in relation to AD-related biological changes. Furthermore, model comparison analyses suggested that inflammatory signatures provided additional statistical information beyond conventional risk factors. However, discrimination between adjacent clinical stages remained modest, indicating that these markers should not yet be considered clinically applicable stratification tools.

Several limitations should be acknowledged. First, the cross-sectional design prevents determination of temporal relationships or causal inference. Longitudinal studies are required to determine whether peripheral inflammatory alterations precede AD-related pathological changes or emerge as consequences of disease progression. Second, although we adjusted for multiple potential confounders, residual confounding cannot be completely excluded. Third, because this was a retrospective exploratory study, the sample size was determined by participant availability rather than an *a priori* power calculation. Statistical power may therefore be limited, particularly for subgroup and interaction analyses, and non-significant findings should be interpreted cautiously. Fourth, inflammatory indices were derived from single blood measurements and may be influenced by transient physiological conditions. Repeated measurements and longitudinal profiling will be necessary to assess their stability over time. Fifth, plasma biomarker analyses were available only for a subset of participants (n = 139), which may limit statistical power and introduce potential selection bias. Finally, this study was conducted in a single-center cohort, and external validation in larger independent populations is warranted to confirm the generalizability of our findings.

In conclusion, this study demonstrates that peripheral hematological inflammatory signatures are associated with clinical severity and biological alterations across the clinical stages across the Alzheimer’s disease continuum. Higher inflammatory burden, particularly the composite inflammatory signature Inflam_PC1, showed incremental associations beyond established demographic and genetic factors and was associated with poorer cognitive and functional performance and higher plasma GFAP levels. These findings suggest that peripheral inflammatory alterations may reflect immune-related biological changes associated with AD heterogeneity, particularly astroglial activation. Longitudinal studies are warranted to determine the temporal relationship between peripheral inflammation and AD-related clinical and biological changes.

## Data Availability

The raw data supporting the conclusions of this article will be made available by the authors, without undue reservation.

## Supplementary material

Supplementary Figure 1 Three ROC curve panels show the discrimination of AD-Aβ+ from CU-Aβ− **(A)**, AD-Aβ+ from MCI-Aβ+ **(B)**, and MCI-Aβ+ from CU-Aβ− **(C)**. Each panel compares the Base, NLR, SII, HRR, All, and PCA models, with sensitivity plotted against 1 − specificity. AUCs with 95% confidence intervals are reported in the legends, and DeLong test P values are shown in the upper left of each panel.

Supplementary Figure 2 Scatterplots show the associations of Aβ40, Aβ42, p-tau181, and NfL with NLR, SII, and HRR across the CU-Aβ−, MCI-Aβ+, and AD-Aβ+ groups. Regression lines with 95% confidence intervals and corresponding correlation coefficients and P values are shown for each group.
