# Supplementary material for: Peripheral hematological inflammatory signatures in the amyloid-defined Alzheimer’s disease continuum: associations with disease stage, cognitive function, and astroglial activation
Source: Front Immunol. 2026 Oct 14;17:1959479. doi: 10.3389/fimmu.2026.1959479 (PMC13713332; doi:10.3389/fimmu.2026.1959479)
Supplement: Supplementary file 1 [file Table1.docx]

| Supplementary Table 1. Brant test results assessing the proportional odds assumption in ordinal logistic regression modelsVariable | **Model 1(Base)** | |  | **Model 2(NLR)** | |  | **Model 3(SII)** | |  | **Model 4(HRR)** | |  | **Model 5(All)** | |  | **Model 6(PCA)** | |
| --- | --- | --- | --- | --- | --- | --- | --- | --- | --- | --- | --- | --- | --- | --- | --- | --- | --- |
|  | χ² | *P* value |  | χ² | *P* value |  | χ² | *P* value |  | χ² | *P* value |  | χ² | *P* value |  | χ² | *P* value |
| Age | 9.25 | 0 |  | 7.84 | 0.01 |  | 7.95 | 0 |  | 9.38 | 0 |  | 7.8 | 0.01 |  | 7.79 | 0.01 |
| Alcohol | 1.92 | 0.17 |  | 2.79 | 0.09 |  | 2.47 | 0.12 |  | 1.85 | 0.17 |  | 2.74 | 0.1 |  | 2.68 | 0.1 |
| APOE ε4 | 0.11 | 0.74 |  | 0.23 | 0.63 |  | 0.1 | 0.75 |  | 0.11 | 0.74 |  | 0.11 | 0.74 |  | 0.18 | 0.68 |
| BMI | 3.92 | 0.05 |  | 3.11 | 0.08 |  | 3.23 | 0.07 |  | 3.89 | 0.05 |  | 2.91 | 0.09 |  | 3.18 | 0.07 |
| Diabetes | 2.05 | 0.15 |  | 2.8 | 0.09 |  | 2.36 | 0.12 |  | 2.07 | 0.15 |  | 2.15 | 0.14 |  | 2.73 | 0.1 |
| Education | 0.36 | 0.55 |  | 0.1 | 0.76 |  | 0.21 | 0.64 |  | 0.38 | 0.54 |  | 0.18 | 0.67 |  | 0.13 | 0.72 |
| Sex-Male | 0.87 | 0.35 |  | 0.69 | 0.41 |  | 0.77 | 0.38 |  | 0.76 | 0.38 |  | 0.4 | 0.53 |  | 0.74 | 0.39 |
| Hyperlipidemia | 2.18 | 0.14 |  | 2.46 | 0.12 |  | 2.69 | 0.1 |  | 2.28 | 0.13 |  | 2.96 | 0.09 |  | 2.59 | 0.11 |
| Hypertension | 0 | 0.94 |  | 0.05 | 0.82 |  | 0.04 | 0.84 |  | 0 | 0.94 |  | 0.11 | 0.74 |  | 0.05 | 0.82 |
| Smoking | 1.4 | 0.24 |  | 1.33 | 0.25 |  | 1.20 | 0.27 |  | 1.41 | 0.23 |  | 1.33 | 0.25 |  | 1.24 | 0.27 |
| NLR |  |  |  | 0.08 | 0.77 |  |  |  |  |  |  |  | 0.38 | 0.54 |  |  |  |
| SII_100 |  |  |  |  |  |  | 0.14 | 0.71 |  |  |  |  | 0.55 | 0.46 |  |  |  |
| HRR |  |  |  |  |  |  |  |  |  | 0.05 | 0.82 |  | 0.13 | 0.72 |  |  |  |
| Inflam_PC1 |  |  |  |  |  |  |  |  |  |  |  |  |  |  |  | 0 | 0.97 |
| Omnibus | 24.13 | 0.01 |  | 23.64 | 0.01 |  | 23.37 | 0.02 |  | 24.42 | 0.01 |  | 24.05 | 0.03 |  | 23.43 | 0.02 |

Supplementary Table 2. Principal component analysis of inflammatory indices for deriving the composite inflammatory index (Inflam_PC1)

| **Principal component** | **Eigenvalue** | **Variance explained (%)** | **Cumulative variance (%)** |  | **Inflammatory index** |
| --- | --- | --- | --- | --- | --- |
| **PC1** | 1.815 | 60.5 | 60.5 |  | NLR \| 0.705; SII \| 0.704; HRR (reversed) \| −0.085 |
| **PC2** | 0.994 | 33.1 | 93.6 |  |  |
| **PC3** | 0.191 | 6.4 | 100.0 |  |  |

Supplementary Table 3 Model performance comparison of ordinal logistic regression models incorporating inflammatory signatures

| Model | AIC | LogLik | Nagelkerke_R2 |
| --- | --- | --- | --- |
| Model 1(Base) | 489.4599 | -232.7299 | 0.3838342 |
| Model 2(NLR) | 473.2255 | -223.6127 | 0.4331343 |
| Model 3(SII) | 471.2147 | -222.6073 | 0.4383676 |
| Model 4(HRR) | 491.1178 | -232.5589 | 0.3847904 |
| Model 5(All) | 473.919 | -221.9595 | 0.4417191 |
| Model 6(PCA) | 471.0071 | -222.5036 | 0.4389057 |

Model definitions: Model 1, baseline model adjusted for age, sex, BMI, education, hypertension, diabetes, hyperlipidemia, smoking, alcohol, and APOE ε4 carrier status; Model 2, baseline + NLR; Model 3, baseline + SII; Model 4, baseline + HRR; Model 5, baseline + NLR + SII + HRR; Model 6, baseline + PCA-derived inflammatory component. AIC: Akaike Information Criterion, lower values indicate better model fit. LogLik: log-likelihood, higher absolute values indicate better fit.

Supplementary Table 4 Calibration assessment of ordinal logistic regression models predicting Alzheimer’s disease continuum stage

| Model | Threshold | Brier | Intercept | Slope | HL_ChiSq | HL_p |
| --- | --- | --- | --- | --- | --- | --- |
| Model 1(Base) | ≥MCI-Aβ^+^ *vs* CU-Aβ^-^ | 0.147 | -3.017 | 5.982 | 5.949 | 0.653 |
|  | ≥AD-Aβ^+^ *vs* CU-Aβ^-^+MCI-Aβ^+^ | 0.184 | -2.226 | 4.471 | 4.025 | 0.855 |
| Model 2(NLR) | ≥MCI-Aβ^+^ *vs* CU-Aβ^-^ | 0.138 | -3.012 | 5.992 | 6.660 | 0.574 |
|  | ≥AD-Aβ^+^ *vs* CU-Aβ^-^+MCI-Aβ^+^ | 0.174 | -2.344 | 4.700 | 6.354 | 0.608 |
| Model 3(SII) | ≥MCI-Aβ^+^ *vs* CU-Aβ^-^ | 0.139 | -2.963 | 5.918 | 6.224 | 0.622 |
|  | ≥AD-Aβ^+^ *vs* CU-Aβ^-^+MCI-Aβ^+^ | 0.173 | -2.359 | 4.723 | 4.801 | 0.779 |
| Model 4(HRR) | ≥MCI-Aβ^+^ *vs* CU-Aβ^-^ | 0.147 | -2.998 | 5.952 | 6.747 | 0.564 |
|  | ≥AD-Aβ^+^ *vs* CU-Aβ^-^+MCI-Aβ^+^ | 0.184 | -2.231 | 4.479 | 8.688 | 0.369 |
| Model 5(All) | ≥MCI-Aβ^+^ *vs* CU-Aβ^-^ | 0.138 | -2.953 | 5.906 | 4.564 | 0.803 |
|  | ≥AD-Aβ^+^ *vs* CU-Aβ^-^+MCI-Aβ^+^ | 0.172 | -2.366 | 4.735 | 4.813 | 0.777 |
| Model 6(PCA) | ≥MCI-Aβ^+^ *vs* CU-Aβ^-^ | 0.138 | -2.970 | 5.932 | 8.528 | 0.384 |
|  | ≥AD-Aβ^+^ *vs* CU-Aβ^-^+MCI-Aβ^+^ | 0.172 | -2.359 | 4.724 | 5.178 | 0.738 |

Calibration was assessed using two cumulative thresholds of the ordinal logistic regression models: ≥MCI-Aβ⁺ *vs* CU-Aβ^-^ and ≥AD-Aβ⁺ *vs* CU-Aβ^-^+MCI-Aβ⁺. Model calibration was evaluated using Brier scores, calibration intercepts, calibration slopes, and the Hosmer-Lemeshow (HL) goodness-of-fit test. Lower Brier scores indicate better calibration, and non-significant HL tests indicate no evidence of model miscalibration.

The base model included age, sex, BMI, education, hypertension, diabetes, hyperlipidemia, smoking, alcohol consumption, and APOE ε4 carrier status. Inflammatory models additionally included NLR, SII, HRR, all three inflammatory indices (All), or the PCA-derived inflammatory component (Inflam_PC1), respectively.

4

Supplementary Table 5 Correlations between peripheral inflammatory indices and cognitive measures

| **Marker** | **Cognitive** | **CU** | **MCI-Aβ⁺** | **AD-Aβ⁺** | **Over all** |
| --- | --- | --- | --- | --- | --- |
| SII | MMSE | -0.185 | -0.329** | -0.187* | -0.233*** |
|  | MoCA | -0.084 | -0.168 | -0.252** | -0.211*** |
|  | ADL | NA | 0.014 | 0.124 | 0.169** |
| NLR | MMSE | -0.250* | -0.241* | -0.078 | -0.248*** |
|  | MoCA | -0.129 | -0.158 | -0.105 | -0.229*** |
|  | ADL | NA | -0.046 | 0.033 | 0.149* |
| HRR | MMSE | -0.086 | -0.049 | 0.029 | 0.139* |
|  | MoCA | -0.069 | -0.095 | -0.004 | 0.134* |
|  | ADL | NA | 0.079 | -0.08 | -0.130* |

**P* < 0.05; ***P* < 0.01; ****P* < 0.001.

Supplementary Table 6 Subgroup analyses of the associations between inflammatory indices and Alzheimer’s disease continuum status

| **Subgroup** | **NLR OR(95%CI)** | ***P*** | **SII (per 100) OR(95%CI)** | ***P*** | **HRR OR(95%CI)** | ***P*** |
| --- | --- | --- | --- | --- | --- | --- |
| **Sex** |  |  |  |  |  |  |
| Male(n=120) | 1.523(1.172-1.978) | 0.002 | 1.284(1.103-1.494) | 0.001 | 0.876(0.656-1.168) | 0.366 |
| Female(n=147) | 1.571(1.076-2.294) | 0.019 | 1.277(1.057-1.542) | 0.011 | 1.067(0.771-1.476) | 0.695 |
| **Age group（years）** |  |  |  |  |  |  |
| Age <65(n=109) | 1.643(1.112-2.429) | 0.013 | 1.340(1.113-1.613) | 0.002 | 0.907(0.659-1.249) | 0.550 |
| Age ≥65(n=158) | 1.363(1.054-1.763) | 0.018 | 1.164(1.000-1.355) | 0.049 | 0.948(0.711-1.265) | 0.719 |
| **APOE ε4 status** |  |  |  |  |  |  |
| APOE ε4 Non-carrier(n=126) | 1.472(1.130-1.918) | 0.004 | 1.247(1.081-1.438) | 0.002 | 0.992(0.722-1.364) | 0.962 |
| APOE ε4 Carrier(n=141) | 1.584(1.119-2.243) | 0.009 | 1.308(1.069-1.600) | 0.009 | 0.900(0.678-1.195) | 0.466 |

Supplementary Table 7 Interaction analyses between inflammatory indices and potential effect modifiers

| Effect modifier | Inflammatory marker | *P* interaction | FDR-adjusted *P* |
| --- | --- | --- | --- |
| Sex | NLR | 0.775 | 0.866 |
|  | SII | 0.857 | 0.866 |
|  | HRR | 0.343 | 0.686 |
| Age group | NLR | 0.167 | 0.500 |
|  | SII | 0.068 | 0.409 |
|  | HRR | 0.866 | 0.866 |
| APOE ε4 status | NLR | 0.807 | 0.890 |
|  | SII | 0.890 | 0.890 |
|  | HRR | 0.581 | 0.890 |
